# Quantitation of Fas and Fas ligand gene expression in human ovarian, cervical and endometrial carcinomas using real-time quantitative RT-PCR

**DOI:** 10.1054/bjoc.2000.1118

**Published:** 2000-04-27

**Authors:** H Das, T Koizumi, T Sugimoto, S Chakraborty, T Ichimura, K Hasegawa, R Nishimura

**Affiliations:** Hyogo Institute of Clinical Research, 13-70 Kitaoji-cho, Akashi, 673-8558, Japan; Gynecology Division, Hyogo Medical Center for Adults, 13-70 Kitaoji-cho, Akashi, 673-8558, Japan

**Keywords:** Fas, FasL, quantitative PCR, primary tumours

## Abstract

Alterations in the expression of Fas (CD95/APO-1) and its ligand (FasL) have been demonstrated in various types of cancers as a mechanism for tumour cell to escape from the immune system. In the present study, we evaluated the expression of the Fas and FasL genes in a wide range of primary gynaecological carcinomas. These included 31 ovarian, 29 cervical and 25 endometrial carcinoma tissues as well as four ovarian and three cervical carcinoma cell lines. Our real-time quantitative reverse transcription polymerase chain reaction analysis revealed that down-regulation of Fas expression is more prominent than the up-regulation of FasL expression in all types of gynaecological cancer studied. This down-regulation of Fas expression was also true for the seven carcinoma cell lines. Only one cervical carcinoma cell line, DoT, exhibited a high level of FasL expression. These results indicated that down-regulation of Fas expression is a common abnormality in many types of cancers including gynaecological cancers, whereas an increase in FasL expression is not a common phenomenon in these cancers. © 2000 Cancer Research Campaign


*British Journal of Cancer* (2000) **82**(10), 1682–1688
© 2000 Cancer Research Campaign
DOI: 10.1054/ bjoc.2000.1118, available online at http://www.idealibrary.com on IDEAL®

### Quantitation of Fas and Fas ligand gene expression in human ovarian, cervical and endometrial carcinomas using real-time quantitative RT-PCR

**H Das[1], T Koizumi[1], T Sugimoto[1], S Chakraborty[1], T Ichimura[2], K Hasegawa[2] and R Nishimura[1,2]**

[1]Hyogo Institute of Clinical Research, 13-70 Kitaoji-cho, Akashi 673-8558, Japan; [2]Gynecology Division, Hyogo Medical Center for Adults, 13-70 Kitaoji-cho, Akashi 673-8558, Japan

**Summary** Alterations in the expression of Fas (CD95/APO-1) and its ligand (FasL) have been demonstrated in various types of cancers as a mechanism for tumour cell to escape from the immune system. In the present study, we evaluated the expression of the Fas and FasL genes in a wide range of primary gynaecological carcinomas. These included 31 ovarian, 29 cervical and 25 endometrial carcinoma tissues as well as four ovarian and three cervical carcinoma cell lines. Our real-time quantitative reverse transcription polymerase chain reaction analysis revealed that down-regulation of Fas expression is more prominent than the up-regulation of FasL expression in all types of gynaecological cancer studied. This down-regulation of Fas expression was also true for the seven carcinoma cell lines. Only one cervical carcinoma cell line, DoT, exhibited a high level of FasL expression. These results indicated that down-regulation of Fas expression is a common abnormality in many types of cancers including gynaecological cancers, whereas an increase in FasL expression is not a common phenomenon in these cancers. © 2000 Cancer Research Campaign

**Keywords**: Fas; FasL; quantitative PCR; primary tumours

Apoptosis or programmed cell death selectively allows certain cells to undergo cell death following many biological signals. Apoptosis plays an important role in development and homeostasis. In malignant cells, these physiological apoptotic pathways are often disturbed, and as a result, they acquire uncontrolled cellular survival and are often resistant to anti-tumour treatment (Thompson et al, 1995).

The Fas (CD95/APO-1) and Fas ligand (FasL) pathway was initially identified as a key system in the regulation of apoptosis within the immune system (Nagata and Golstein, 1995). FasL with its receptor, Fas, induces apoptosis in sensitized cells. This Fas-mediated apoptosis is involved in thymocyte clonal deletion and tolerance acquisition (Yonehara et al, 1994), T-cell activation-induced cell death (Alderson et al, 1995), immune response termination (Daniel and Krammer, 1994) and T-cell-mediated cytotoxicity (Ju et al, 1994). The Fas/FasL system has also been reported to have roles in the maintenance of immune privilege in mouse testis (Bellgrau et al, 1995) and in the anterior chamber of the eye (Griffith et al, 1995). FasL expressed in these tissues induces apoptosis in activated lymphocytes that infiltrate these sites.

In addition to being expressed in the immune system, Fas is constitutively expressed by a broad panel of normal epithelial cells (Leithauser et al, 1993). Although the roles of the Fas system in non-lymphoid tissues are not thoroughly understood, a role in the maintenance of physiological cell turnover has been suggested (Keane et al, 1996; Suzuki et al, 1996). Fas expression and response to it are variable in human lymphoid (Debatin and Krammer, 1995; Shima et al, 1995) and non-lymphoid malignancies (Owen-Schaub et al, 1994). Several studies have reported decreased levels of Fas in cancer cells, and the resultant resistance to Fas-mediated apoptosis may contribute to their escape from the immune system (Leithauser et al, 1993; Moller et al, 1994; Keane et al, 1996; Strand et al, 1996; Gratas et al, 1998). Furthermore, recent studies have provided evidence that FasL expression is found in various types of tumour cells. O'Connell et al (1996) have proposed that FasL-expressing tumour cells counterattack cytotoxic T-cells, which provides another mechanism by which tumours can escape from the immune system.

Proliferation and regression of gynaecological tissues such as ovary, cervix and endometrium is hormone-dependent, and Fas is constitutively expressed in these tissues (Leithauser et al, 1993). Thus, it would be interesting to know whether the expression of Fas and/or FasL is also altered in these gynaecological cancers. Although there have been reports concerning the expression of these genes in gynaecological cancers, quantitative measurement of the gene expression was not performed in those studies (Imai et al, 1997; Takagi et al, 1998). Here, we investigated the relative expression of Fas and FasL in human gynaecological carcinomas of the ovary, cervix and endometrium using a recently developed, highly sensitive quantitative real-time polymerase chain reaction (PCR) system (Gibson et al, 1996).

### MATERIALS AND METHODS

### Tissue samples and cell lines

Expression of Fas and FasL genes was examined in 31 ovarian, 29 cervical and 25 endometrial carcinoma tissues, nine ovarian, six cervical and eight endometrial normal tissues, and four ovarian

*Received 17 February 1999*
*Revised 6 January 2000*
*Accepted 3 February 2000*

*Correspondence to:* T Koizumi and three cervical carcinoma cell lines. Surgically resected tumour specimens were obtained from the Hyogo Medical Center for Adults adjoining to our Institute. The tumours were classified by the Histo-Pathology Department of the Center according to the WHO guidelines and staged according to the TNM classification of malignant tumours defined by the International Union Against Cancer. Normal tissue samples were collected from the site on the body that was most distant from the tumours or from different patients resected for a non-cancerous reason. All samples were obtained with informed consent. Collected tissue samples were snap-frozen in liquid nitrogen immediately after resection and stored at –80°C unless used. Cervical carcinoma cell lines, HeLa and CaSki, and T lymphoma cell line, Jurkat were obtained from ATCC (Rockville, MD, USA). Another cervical carcinoma cell line, DoT, was a gift of Dr R A Pattillo, Medical College of Wisconsin, Wisconsin, USA. Ovarian carcinoma cell lines, RMG-I, RMG-S, RTSG, RMUG-L, were gifts of Dr S Nozawa, Keio University School of Medicine, Tokyo, Japan.

### Standard RT-PCR for Fas and FasL mRNA

Total RNA was extracted from the frozen tissues and cultured cell lines using TRIzol (Life Technologies, Inc., Gaithersburg, MD, USA). One microgram of each total RNA was reverse transcribed using a random hexamer and a SuperScript pre-amplification system (Life Technologies). An aliquot of 1/20th of the resulting cDNA was used for a PCR amplification. Fas primers were Fas I: CAGAACTTGGAAGGCCTGCATC and Fas II: TCTGTTCT-GCTGTGTCTTGGAC (O'Connell et al, 1996) which amplified a fragment of 682 bp. Specific primers were FasL I: ACACCTATG-GAATTGTCCTGC and FasL II: GACCAGAGAGAGCTCA-GATACG, which amplified a fragment of 311 bp, and GAPDH I: CATCACCATCTTCCAGGAGC and GAPDH II: GGATGAT-GTTCTGGAGAGCC, which amplified a fragment of 404 bp. These four primers were designed using the Gene Works software program (IntelliGenetics, Inc., Mountain View, CA, USA). The cDNA was amplified in 50 μl of PCR buffer containing dNTP, magnesium chloride ($MgCl_2$), using Ex-Taq DNA polymerase (TaKaRa, Shuzo, Kyoto, Japan), and in a Peltier thermal cycler (PTC-200; MJ Research, MA, USA). The annealing temperatures for the specific primers, were 55°C for FasL and Fas, and 60°C for glyceraldehyde 3-phosphate dehydrogenase (GAPDH). The number of amplification cycles were 35 for FasL, 40 for Fas and 25 for GAPDH. The other conditions were the same, with initial denaturation for 3 min at 94°C, cycle denaturation for 10 s at 92°C, annealing for 30 s and extension at 72°C for 60 s. A 10 μl aliquot of amplified product was then analysed on a 2% Metaphor agarose gel. Visualization and photography were performed with the ImageMaster VDS (Pharmacia Biotech, Uppsala, Sweden), a video documentation system for gel electrophoresis after ethidium bromide staining.

### Real-time quantitative PCR for Fas and FasL mRNA

To evaluate the expression of Fas and FasL, quantitative PCR was performed using Sequence Detector (ABI PRISM 7700, P E Applied Biosystems, Foster City, CA, USA). Selection of primers and probes for Fas, FasL and GAPDH were performed by using Primer Express software (P E Applied Biosystems). Selected forward (F) and reverse (R) primers, and probes are as follows; Fas F: 5′ TGAAGGACATGGCTTAGAAGTG 3′, Fas R: 5′ GGT-GCAAGGGTCACAGTGTT 3′, Fas probe: 5′-(FAM)- AAACTG-CACCCGGACCCAGAATACC-(TAMRA)-3′, FasL F: 5′ GCAG-CCCTTCAATTACCCAT 3′, Fasl R: 5′ CAGAGGTTGGACA-GGGAAGAA 3′, FasL probe: 5′-(FAM)-TCCCCAGATCTAC-TGGGTGGACAGC-(TAMRA)-3′, GAPDH F: 5′ GAAGGTGA-AGGTCGGAGTC 3′, GAPDH R: 5′ GAAGATGGTGATGG-GATTTC 3′, GAPDH probe: 5′-(FAM)-CAAGCTTCCCGT-TCTCAGCC-(TAMRA)-3′. These primer sets amplify 118 bp of Fas, 101 bp of FasL, and 226 bp of GAPDH cDNA fragment respectively. The TaqMan probes were used, which consisted of an oligonucleotide with a 5′ FAM (6-carboxy-fluorescein) reporter dye and 3′ quencher dye, TAMRA (6-carboxy-tetramethyl-rhodamine). A portion (1/20) of each cDNA was used for quantitative PCR in a 50 μl volume using Master Mix, which includes PCR buffer, $MgCl_2$, dATP, dCTP, dGTP, dUTP, AmpErase UNG and AmpliTaq Gold DNA polymerase (P E Applied Biosystems). Thermal cycling conditions were an initial 2 min at 50°C and 10 min at 95°C, as recommended by the manufacturer. Cycle conditions were 15 s at 95°C and 1 min at 60°C. For Fas and GAPDH expression, 40 cycles of amplification were performed and 50 cycles were performed for FasL expression.

Real-time quantitation was done based upon the TaqMan assay using a fluorogenic oligonucleotide probe labelled with both a fluorescent dye and a quencher dye according to manufacturer's instruction. In the intact TaqMan probe, the 5′ fluorescent reporter dye was quenched by the 3′ quencher dye through a Foster-type energy transfer. Fluorogenic DNA probes (TaqMan probes) were hydrolysed during PCR upon hybridization to the template DNA by *Taq* DNA polymerase, whose nuclease activity is dependent on the 5′ secondary structure. After hydrolysis, the release of the reporter signal caused an increase in fluorescence intensity that was proportional to the accumulation of the PCR product. The fluorescence intensity of the reporter label was normalized using the rhodamine derivative ROX as a passive reference label present in the buffer solution. The system generates a real-time amplification plot based upon the normalized fluorescence signal. Subsequently the threshold cycle (CT), i.e. the fractional cycle number at which the amount of amplified target reached a fixed threshold was determined. The CT was then used for kinetic analysis and was proportional to the initial number of target copies in the sample. The starting quantity of a sample was calculated after comparison of the CTs of a serial dilution of a positive control. Figure 1 showed the amplification plots (left panel) and the standard curves (right panel) of serially diluted positive controls for Fas (1- to 128-fold), FasL (1- to 16 384-fold), and GAPDH (1- to 64-fold) respectively. Jurkat was used as a positive control for Fas expression, and DoT was used as a postive control for FasL and GAPDH expression. Starting quantity of most diluted positive control was defined as one.

### Statistical analyses

Differences between expression levels of Fas or FasL among the cancer patient group and the normal group of each type (ovarian, cervical, endometrial) and also among the normal groups were determined following the Mann–Whitney test using StatView software (Abacus Concepts, Inc., Berkeley, CA, USA). The mean values and standard deviations were presented in the respective figure legends. Statistical significance was accepted when $P < 0.05$.

© 2000 Cancer Research Campaign

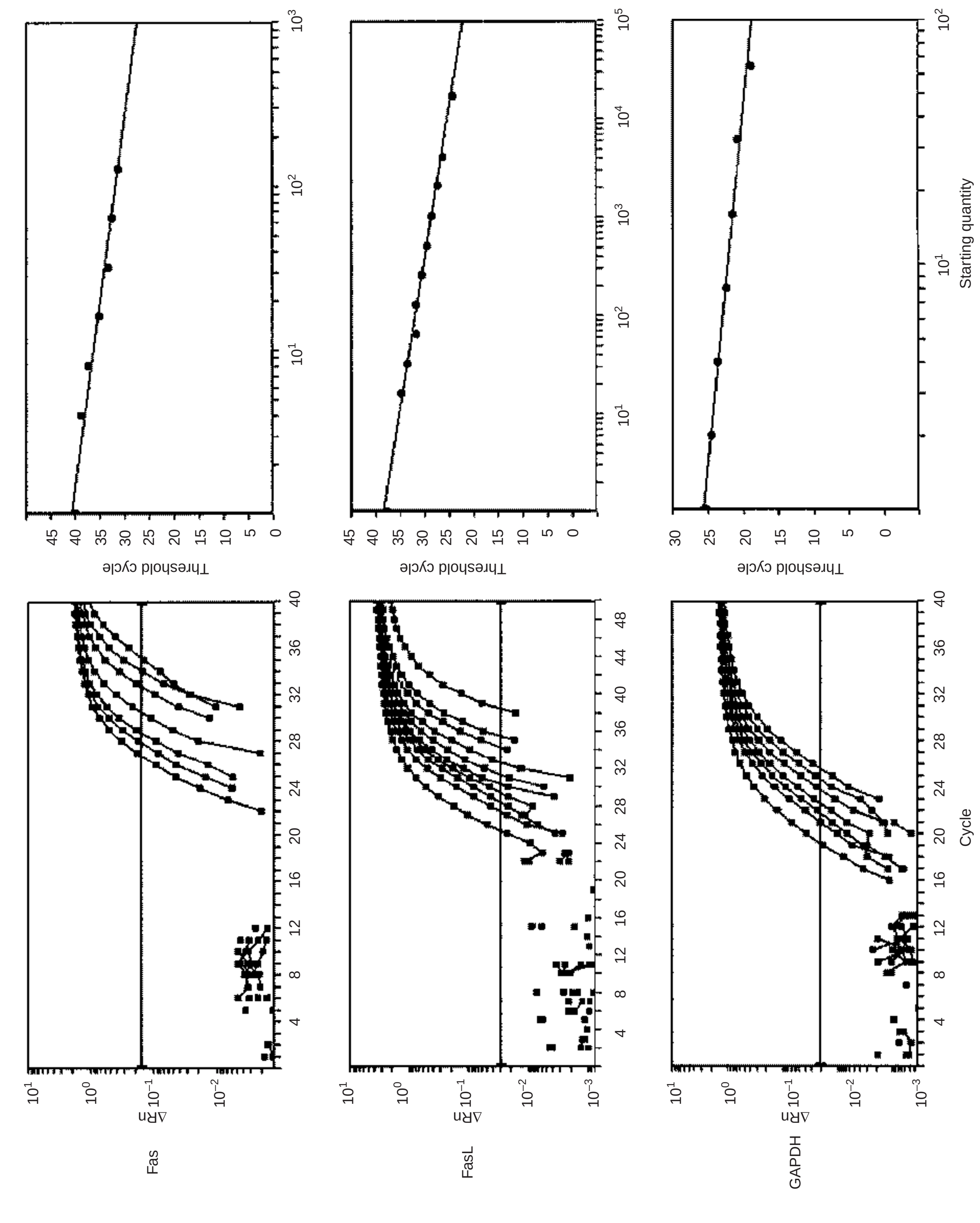

**Figure 1** PCR product detection in real time. Amplification plots (left) and standard curves (right) for Fas, FasL, and GAPDH expression. Twofold seial dilutions of positive control cDNA were amplified using the 5′ nuclease assay and the sequence detector. Jurkat was used as a positive control for Fas, and DoT was used as a positive control for FasL and GAPDH

*© 2000 Cancer Research Campaign*

### RESULTS

### Fas and FasL mRNA expression in ovarian cancer tissues

We first analysed expression of Fas and FasL gene expression along with GAPDH in ovarian cancer tissues by the standard RT-PCR procedure (Figure 2). Many of the cancer tissues showed decreased levels of Fas gene expression when the levels were compared with those in normal ovarian tissues. The levels of FasL gene expression varied among the samples. To obtain more accurate data, we utilized the recently developed real-time quantitative assay. Based on the standard curves which had the large dynamic ranges for Fas (1–128), FasL (1–16 384), and GAPDH (1–64) expression levels (Figure 1), Fas and FasL expression levels in the samples were plotted after the levels were corrected with GAPDH expression levels (Figure 3). In the primary ovarian cancer group ($n$ = 31), the levels of Fas expression were significantly decreased ($P$ < 0.001) compared with those in the normal group ($n$ = 9). The levels of FasL gene expression varied among the samples and showed no significant difference when compared with those in normal tissues. Decreased levels of Fas and FasL expression were observed in the four ovarian carcinoma cell lines.

### Fas and FasL mRNA expression in cervical cancer tissues

A significant decrease in the levels of Fas gene expression were also observed ($P$ < 0.04) in the cervical carcinomas tissues ($n$ = 29) compared with those in the normal tissues ($n$ = 6) (Figure 4). Various levels of FasL gene expression were found in different tissue samples. Two cancer tissues showed clearly increased levels of FasL expression and some showed decreased levels. However, no significant difference was found between the cancerous group ($P$ = 0.7593) and the respective normal group. Among the three cervical carcinoma cell lines studied, only one (DoT) showed a higher level of FasL, while the other two (HeLa and CaSki) did not show any increase compared with the normal tissues.

### Fas and FasL mRNA expression in endometrial cancer tissues

Among 25 endometrial carcinoma samples evaluated with eight normal tissues, levels of Fas gene expression were found to be significantly decreased ($P$ < 0.003) (Figure 5) as like as ovarian and cervical samples. No significant difference ($P$ = 0.2231) in the levels of FasL gene expression was found between the cancerous group and the normal group. Only one cancer tissue sample showed a clearly increased level of FasL and some showed decreased levels.

### Comparison of Fas and FasL expression in normal ovarian, cervical and endometrial tissues

Our results show that all the normal ovarian, cervical and endometrial tissues express certain levels of Fas and FasL (Figures 3–5). The levels of Fas expression were not different among these tissues while the levels of FasL expression in normal endometrial tissues were about ninefold higher than those in the normal ovarian tissues and about 12-fold higher than those in normal cervical tissues.

### DISCUSSION

RT-PCR assay is an excellent method to assess gene expression due to its great sensitivity. Although PCR has provided a powerful tool, it is imperative that it be used properly for quantitation. One approach measures PCR product quantity in the log phase of the reaction before the plateau (Kellogg et al, 1990). Another method, quantitative competitive PCR, has been developed and is used

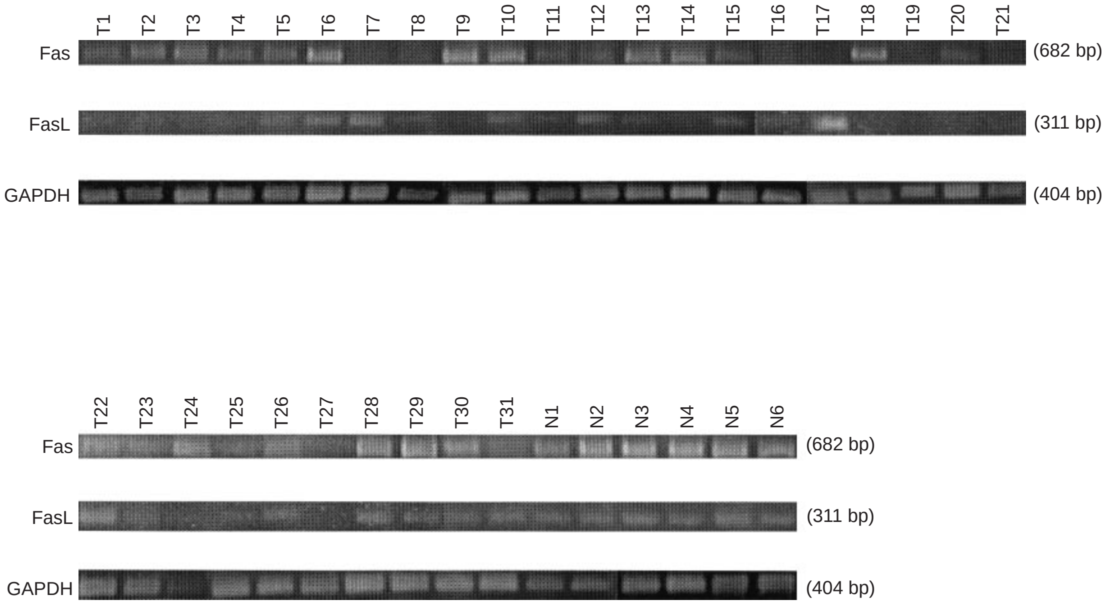

**Figure 2** Standard RT-PCR analysis of Fas and FasL expression in ovarian tumour (T) and normal (N). The corresponding expression of GAPDH is shown in the lower panel

*© 2000 Cancer Research Campaign* 

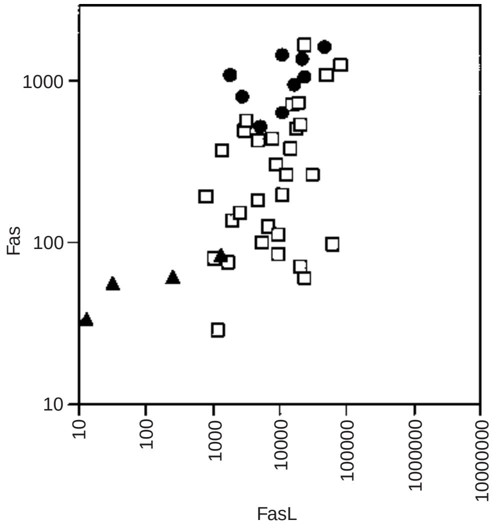

**Figure 3** Real-time quantitative PCR analysis of Fas and FasL expression in tumour (■) and normal (●) tissues, and cell lines (▲) of ovarian origin. Statistical analyses of Fas; normal samples ($n$ = 9) mean ± s.d.= 1053.7 ± 375.4, and tumour samples (n = 31) mean ± s.d.= 376.6 ± 385.4, $P$-values between tumour and normal is $P < 0.001$, and FasL; normal samples mean ± s.d.= 15200.0 ± 13830.1, and tumour samples mean ± s.d.= 15046.4 ± 18257.7, $P = 0.5490$

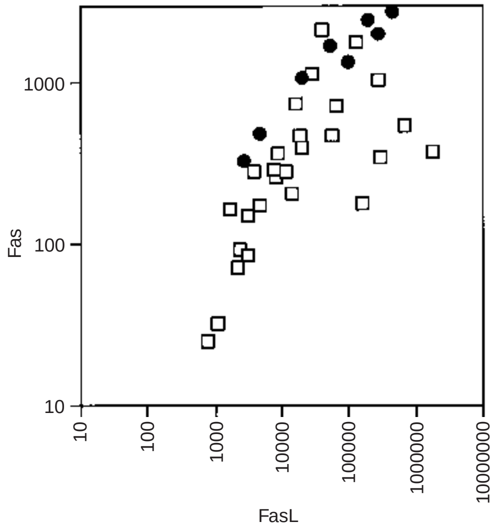

**Figure 5** Relative expression of Fas and FasL in tumour (□) and normal (●) tissues of endometrial origin corrected by GAPDH expression after real-time quantitative PCR. Statistical analyses of Fas; normal samples ($n$ = 8) mean ± s.d.= 1495.8 ± 882.5, and tumour samples ($n$ = 25) mean ± s.d.= 490.4 ± 518.8, $P$-values between tumour and normal is $P < 0.003$, and FasL; normal samples mean ± s.d.= 134970.3 ± 155817.8, and tumour samples mean ± s.d.= 143601.6 ± 366602.2, $P = 0.2231$

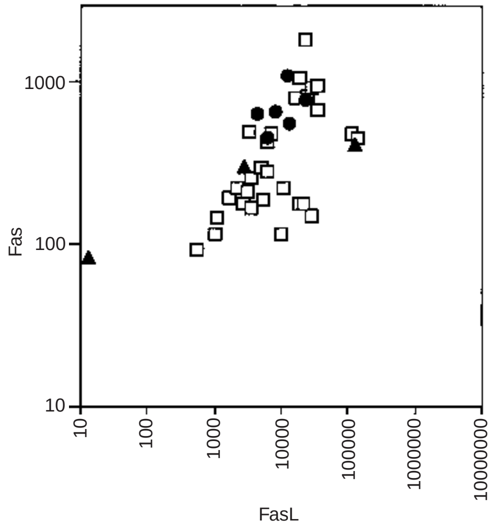

**Figure 4** Relative expression of Fas and FasL in tumour (■) and normal (●) tissues, and cell lines (▲) of cervical origin corrected by GAPDH expression after real-time quantitative PCR. Statistical analyses of Fas; normal samples ($n$ = 6) mean ± s.d.= 696.1 ± 228.4, and tumour samples ($n$ = 29) mean ± s.d.= 432.9 ± 392.9, $P$-values between tumour and normal is $P < 0.04$, and FasL; normal samples mean ± s.d.= 11371.3 ± 6788.6, and tumour samples mean±s.d.= 19730.6 ± 31268.5, $P = 0.7593$

widely for PCR quantitation (Becker-Andre, 1991). Both methods, however, are very time-consuming and cannot be used in a routine setting. The final results sometimes vary, which make them hard to assess and the use of the competitor DNA molecule in the competitive PCR is a potential source of contamination. In contrast, DNA amplification by the new method is detected in a closed tube, and no post-PCR sample handling is necessary, thus minimizing cross-sample contamination (Gibson et al, 1996). The instrument provides real-time quantitative information and has a very large dynamic range of starting target molecule determination (up to five orders of magnitude). The same system has been used successfully in the monitoring of leukaemia cells expressing chimaeric genes (Mensink et al, 1998) and the measurement of cytokine gene expression in lymphocytes (Rissoan et al, 1999).

With the advantage of real-time quantitative RT-PCR analysis in this study, we demonstrated a statistically significant decrease in the expression of Fas gene in the gynaecological carcinomas compared with the respective normal tissues. Although Imai et al (1997) have demonstrated frequent expression of Fas in endometrial and ovarian carcinomas, they did not show any quantitative comparison with normal tissues. Our findings correlate with earlier reports of decreased levels of Fas in primary breast carcinomas (Keane et al, 1996), oesophageal carcinomas (Gratas et al, 1998), hepatocellular carcinomas (Strand et al, 1996), colon carcinomas (Moller et al, 1994) and lung carcinomas (Leithauser et al, 1993). Thus, it appears that a loss or decrease in Fas gene expression may be one of the common changes that occur in a wide variety of cancers. Recently, negative correlation of mutant Ras or p53 expression with Fas gene expression has been reported in vitro culture systems (Sheard et al, 1997; Fenton et al, 1998; Muller

*© 2000 Cancer Research Campaign*

et al, 1998). Further studies are required to answer the interesting question of whether the decreased expression of the Fas gene correlates with these major oncogenic processes in primary cancers.

The resultant loss of Fas may make it easier for tumours to survive and grow in spite of various anti-tumour mechanisms in vivo (Zornig et al, 1995). The escape of Fas-negative tumours from immune-attack by anti-tumour lymphocytes has been experimentally demonstrated (Zornig et al, 1995). Tumour cells with decreased levels of Fas may be resistant to cytotoxic drugs (Micheau et al, 1997). Recently, it has been shown that apoptosis induced by c-*myc* oncoprotein is dependent on Fas expression (Hueber et al, 1997). Accordingly, the loss of Fas expression may result in the resistance of c-*myc*-transformed tumour cells to apoptosis and contribute to tumour growth.

Up-regulation of FasL expression was also reported in melanomas (Hahne et al, 1996), glioblastomas (Saas et al, 1997), hepatocellular carcinomas (Strand et al, 1996), lung carcinomas (Niehans et al, 1997), liver metastases of colon adenocarcinomas (Shiraki et al, 1997), colon carcinoma cell lines (O’Connell et al, 1996) and oesophageal carcinoma (Gratas et al, 1998). O’Connell et al (1996) have proposed a hypothesis in which immune cells are counterattacked by FasL-bearing tumours. Our analysis of primary ovarian, endometrial and cervical carcinomas revealed a wide range in the expression of FasL and only some of cervical and endometrial carcinoma tissues demonstrated an up-regulation of FasL expression. No up-regulation of FasL in endometrial and ovarian carcinomas has also been reported recently (Takagi et al, 1998). Thus, the hypothesis in which immune cells are counterattacked by FasL-bearing tumour cells may not be generally applicable in these gynaecological cancers.

Fas expression was demonstrated in the normal ovary, endometrium and breast epithelial cells, where menstrual cycle-dependent rounds of proliferation and regression due to apoptosis were found (Leithauser et al, 1993; Guo et al, 1994; Tabibzadeh et al, 1995). Levels of Fas expression were not very different among these tissues. However, the level of FasL expression was much higher in the endometrium compared with ovary and cervix (about ninefold higher than in the ovary, and 12-fold higher than in the cervix). FasL expression was also reported in normal uterine tissues but not in ovarian tissues by RT-PCR and Western blot analysis (Xerri et al, 1997). Detection of FasL in ovarian tissues in our study may be only due to the greater sensitivity of our assay system. The higher levels of FasL observed in normal endometrial tissues compared with ovarian and cervical tissues appear to be related to its greater menstrual cycle-dependent tissue proliferation and regression.

In summary, we demonstrated a significant decrease in Fas expression in gynaecological cancer tissues but not an increase in FasL expression that was reported earlier in other carcinomas. We found that only a limited number of our samples exhibited an increase in FasL expression. However, since we analysed the gene expression in the RNA extracted from blocks of tumour tissues which may contain various percentages of tumour cells and normal cells, it is possible that the obtained results may not really reflect the expression of these genes in tumour cells. Especially, the presence of activated lymphocytes expressing large amounts of Fas and FasL may affect the results. Combination of these results with further studies using other techniques such as in situ hybridization will be necessary to clarify this point.

### ACKNOWLEDGEMENTS

This work was supported in part by a research grant from the Hyogo prefectural government, Hyogo, Japan. The authors are grateful to Ms I Ushio and Ms K Miki for their excellent technical assistance.

### REFERENCES

Alderson MR, Tough TW, Davis-Smith T, Braddy S, Falk B, Schooley KA, Goodwin RG, Smith CA, Ramsdell F and Lynch DH (1995) Fas ligand mediates activation induced cell death in human T lymphocytes. *J Exp Med* **181**: 71–77

Becker-Andre M (1991) Quantitative evaluation of mRNA levels. *Meth Mol Cell Biol* **61**: 3724–3728

Bellgrau D, Gold D, Selawry H, Moore J, Franzusoff A and Duke RC (1995) A role for CD95 ligand in preventing graft rejection. *Nature (Lond.)* **377**: 630–632

Daniel PT and Krammer PH (1994) Activation induces sensitivity toward APO-1 (CD95)-mediated apoptosis in human B cells. *J Immunol* **152**: 5624–5632

Debatin K-M and Krammer PH (1995) Resistance to APO-1 (CD95) induced apoptosis in T-ALL is determined by a *bcl*-2 independent anti-apoptotic program. *Leukemia* **9**: 815–820

Evan G and Littlewood T (1998) A matter of life and cell death. *Science* **281**: 1317–1321

Fenton RG, Hixon JA, Wright PW, Brooks AD and Sayers TJ (1998) Inhibition of Fas (CD95) expression and Fas-mediated apoptosis by oncogenic Ras. *Cancer Res* **58**: 3391–3400

Gibson UEM, Heid CA and Williams PM (1996) A novel method for real time quantitative RT-PCR. *Genome Res* **6**: 995–1001

Gratas C, Tohma Y, Barnas C, Taniere P, Hainaut P and Ohgaki H (1998) Up-regulation of Fas (APO-1/CD95) ligand and down-regulation of Fas expression in human esophageal cancer. *Cancer Res* **58**: 2057–2062

Griffith TS, Brunner T, Fletcher SM, Green DR and Ferguson TA (1995) Fas ligand-induced apoptosis as a mechanism of immune privilege. *Science* **270**: 1189–1192

Guo MW, Mori E, Xu JP and Mori T (1994) Identification of Fas antigen associated with apoptotic cell death in murine ovary. *Biochem Biophys Res Commun* **203**: 1438–1446

Hahne M, Rimoldi D, Schroter M, Romero P, Schreier M, French LE, Schneider P, Bornand T, Fontana A, Lienard D, Cerottini J-C and Tschopp J (1996) Melanoma cell expression of Fas (Apo-1/CD95) ligand: implications for tumor immune escape. *Science* **274**: 1363–1366

Hueber A-O, Zoring M, Lyon D, Suda T, Nagata S and Evan GI (1997) Requirement for the CD95 receptor-ligand pathway in c-*Myc*-induced apoptosis. *Science* **278**: 1305–1309

Imai A, Horibe S, Takagi A, Ohno T and Tamaya T (1997) Frequent expression of Fas in gonadotropin-releasing hormone receptor-bearing tumors. *Eur J Obstet Gynecol Reprod Biol* **74**: 73–78

Ju ST, Cui H, Panka DJ, Ettinger R and Marshak-Rothstein A (1994) Participation of target Fas protein in apoptosis pathway induced by CD4+ Th1 and CD8+ cytotoxic T cells. *Proc Natl Acad Sci USA* **91**: 4185–4189

Keane MM, Ettenberg SA, Lowrey GA, Russell EK and Lipkowitz S (1996) Fas expression and function in normal and malignant breast cell lines. *Cancer Res* **56**: 4791–4798

Kellogg DE, Sninsky JJ and Kowk S (1990) Quantitation of HIV-1 proviral DNA relative to cellular DNA by the polymerase chain reaction. *Anal Biochem* **189**: 202–208

Leithauser F, Dhein J, Mechtersheimer G, Koretz K, Bruderlein S, Henne C, Schmidt A, Debatin K-M, Krammer PH and Moller P (1993) Constitutive and induced expression of APO-1, a new member of the nerve growth factor/tumor necrosis factor receptor superfamily, in normal and neoplastic cells. *Lab Invest* **69**: 415–429

Mensink E, Locht AV, Shattenberg A, Linders E, Schaap E, Kessel AG and Whitte TD (1998) Quantitation of minimal residual disease on Philadelphia chromosome positive chronic myeloid leukemia patients using real-time quantitative RT-PCR. *Br J Haematol* **102**: 768–774

Micheau O, Solary E, Hammann A, Martin F, Dimanche-Boitrel MT (1997) Sensitization of cancer cells treated with cytotoxic drugs to fas-mediated cytotoxicity. *J Natl Cancer Inst* **89**: 783–789

© 2000 Cancer Research Campaign 

Moller P, Koretz K, Leithhauser F, Bruderlein S, Henne C, Quentmeir A and Krammer PH (1994) Expression of APO-1 (CD95), a member of the NGF/TNF receptor superfamily, in normal and neoplastic colon epithelium. *Int J Cancer* **57**: 371–377
Muller M, Wilder S, Bannasch D, Israeli D, Lehlbach K, Li-Weber M, Friedman SL, Galle PR, Stremmel W, Oren M and Krammer PH (1998) p53 activates the CD95 (APO-1/Fas) gene in response to DNA damage by anticancer drugs. *J Exp Med* **188**: 2033–2045
Nagata S and Golstein P (1995) The Fas death factor. *Science* **267**: 1449–1456
Niehans GA, Brunner T, Frizelle SP, Liston JC, Salerno CT, Knapp DJ, Green DR and Kratzke RA (1997) Human lung carcinomas express Fas ligand. *Cancer Res* **57**: 1007–1012
O'Connell J, O'Sullivan GC, Collins JK and Shanahan F (1996) The Fas counterattack: Fas-mediated T cell killing by colon cancer cells expressing Fas ligand. *J Exp Med* **184**: 1075–1082
Owen-Schaub LB, Radinsky R, Kruzel E, Berry K and Yonehara S (1994) Anti-Fas on nonhematopoietic tumors: levels of Fas/APO-1 and *bcl-2* are not predictive of biological responsiveness. *Cancer Res* **54**: 1580–1586
Rissoan M, Soumelis V, Kadowaki N, Grouard G, Briere F, Malefyt R and Liu Y (1999) Reciprocal control of T helper cell and dendritic cell differentiation. *Science* **283**: 1183–1186
Sheard MA, Vojtesek B, Janakova L, Kovarik J and Zaloudik J (1997) Up-regulation of Fas (CD95) in human p53 wild-type cancer cells treated with ionizing radiation. *Int J Cancer* **73**: 757–762
Shima Y, Nishimoto N, Ogata A, Fujii Y, Yoshizaki K and Kishimoto T (1995) Myeloma cells express Fas antigen/APO-1 (CD95) but only some are sensitive to anti-Fas antibody resulting in apoptosis. *Blood* **85**: 757–764
Shiraki K, Tsuji N, Shioda T, Isselbacher KJ and Takahashi H (1997) Expression of Fas ligand in liver metastases of human colonic adenocarcinomas. *Proc Natl Acad Sci USA* **94**: 6420–6425
Strand S, Hofmann WJ, Hug H, Muller M, Otto G, Strand D, Mariani SM, Stremmel W, Krammer PH and Galle PR (1996) Lymphocyte apoptosis induced by CD95 (APO-1/Fas) ligand-expressing tumor cells: a mechanism of tumor evasion? *Nat Med* **2**: 1361–1366
Suzuki A, Enari M, Eguchi Y, Matsuzawa A, Nagata S, Tsujimoto Y and Iguchi T (1996) Involvement of Fas in regression of vaginal epithelia after ovariectomy and during an estrous cycle. *EMBO J* **15**: 211–215
Tabibzadeh S, Zupi E, Babaknia A, Liu R, Marconi D and Romanini C (1995) Site and menstrual cycle-dependent expression of proteins of the tumour necrosis factor (TNF) receptor family, and BCL-2 oncoprotein and phase-specific production of TNF-α in human endometrium. *Hum Reprod* **10**: 277–286
Takagi A, Imai A, Horibe S, Ohno T and Tamaya T (1998) Lack of evidence for expression of Fas ligand in Fas-bearing tumors. *Oncol Rep* **5**: 377–380
Thompson CB (1995) Apoptosis in pathogenesis and genes and treatment of disease. *Science* **267**: 1456–1461
Xerri L, Devilard E, Hassoun J, Mawas C and Birg F (1997) Fas ligand is not only expressed in immune privileged human organs but is also coexpressed with Fas in various epithelial tissues. *Mol Pathol* **50**: 87–91
Yonehara S, Nishimura Y, Kishil S, Yonehara M, Takazawa K, Tamatani T and Ishii A (1994) Involvement of apoptosis antigen Fas in clonal deletion of human thymocytes. *Int Immunol* **6**: 1849–1856
Zornig M, Grzeschiczek A, Kowalski M-B, Hartmann K-U and Moroy T (1995) Loss of Fas/APO-1 receptor accelerates lymphomagenesis in Eμ L-*myc* transgenic mice but not in animals infected with MoMuLV. *Oncogene* **10**: 2397–2401

*© 2000 Cancer Research Campaign*
